# Serum Sclerostin Levels in Patients with Ankylosing Spondylitis and Rheumatoid Arthritis: A Systematic Review and Meta-Analysis

**DOI:** 10.1155/2017/9295313

**Published:** 2017-05-03

**Authors:** Jianfeng Shi, Haijian Ying, Juping Du, Bo Shen

**Affiliations:** ^1^Wenzhou Medical University, Wenzhou, Zhejiang, China; ^2^Department of Clinical Laboratory, Taizhou Hospital, Wenzhou Medical University, Zhejiang, China

## Abstract

*Objective.* Current studies of serum sclerostin levels in AS and RA patients are inconsistent. This meta-analysis was performed to identify the association of serum sclerostin level with AS and RA patients.* Methods.* Embase, PubMed, MEDLINE, and Cochrane Library databases (up to 25 January 2017) were used to collect all relevant published articles. Studies were pooled and standard mean difference (SMD) with 95% confidence interval (CI) was calculated. All data analyses were performed using RevMan 5.3.* Results.* Totally eight studies of AS including 420 AS patients and 317 healthy controls (HC) and three studies of RA including 145 RA patients and 127 HC were finally included in this meta-analysis. The results revealed that the serum sclerostin levels in both AS patients (SMD = −0.14; 95% CI = [−0.39,0.11]; *P* = 0.28) and RA patients (SMD = −0.10; 95% CI = [−0.34,0.15]; *P* = 0.43) were not significantly different from those in HC.* Conclusion.* The difference of serum sclerostin levels in AS and RA patients was not significantly different from HC, indicating that the sclerostin may not associate with the development of AS and RA.

## 1. Introduction

Ankylosing spondylitis (AS) and rheumatoid arthritis (RA) are both chronic systemic autoimmune diseases. AS is a chronic inflammatory disease that mainly involves axial skeleton and sacroiliac joint, which is characterized by entheses inflammation, resulting in uncontrolled osteoproliferation that usually leads to fusion and rigidity of the affected spine. RA is characterized by persistent inflammation of synovium, which eventually gives rise to joint destruction and deformation [1]. Progress had been greatly made in the long-term research; however, the pathogenesis of AS and RA is still unclear. Recently, several studies had revealed that Wnt signaling pathway inhibitor sclerostin plays a significant role in the development of AS and RA [2, 3].

Sclerostin is encoded by SOST gene and mainly expressed and secreted by osteocytes and other terminally differentiated cells embedded within mineralized matrix, such as osteocytes, chondrocytes, and cementocytes [4]. Sclerostin emerges as a natural inhibitor regulating the Wnt/*β*-catenin pathway, which had been considered as a crucial modulating pathway for bone formation [5]. Substantially, the canonical signaling was activated by the binding of Wnt ligands to the Frizzled receptor and the coreceptors low-density lipoprotein receptor-related proteins 5 and 6 (LRP-5 and LRP-6), thus maintaining the structural stability of *β*-catenin, which acts as a prominent component in the signaling pathway. Subsequently, *β*-catenin increases in cytoplasm and translocates into nucleus to modulate target genes transcription [6]. Sclerostin is capable of binding to LRP-5/LRP-6, which prevents Wnt proteins from reaching LRP-5/LRP-6, resulting in the inhibition of the canonical Wnt signaling pathway [7, 8].

The serum sclerostin levels had been suggested to implicate the pathogenesis of AS and RA in several studies; however, the results were inconsistent [9]. Therefore, the objective of our study is to comprehensively estimate the role of serum sclerostin in the development of AS through a meta-analysis.

## 2. Materials and Methods

### 2.1. Publication Selection

This meta-analysis was performed using PubMed, Embase, MEDLINE, and Cochrane Library databases to identify all relative publications involved in serum sclerostin level in AS. The search keywords were as follows: “sclerostin,” “SOST,” “ankylosing spondylitis,” “spondyloarthritis,” “axial spondyloarthritis,” “peripheral spondyloarthritis,” “radiographic axial spondyloarthritis,” “non-radiographic axial spondyloarthritis,” “Bechterew's disease,” “rheumatoid Arthritis,” and “RA.” Studies meeting the criteria as follows were included: (1) they were case-control studies or section-control studies; (2) study subjects were human AS patients according to the modified New York criteria [10] or ASAS diagnosis and classification criteria [11]; (3) studies provided the mean and the standard deviation (mean ± SD) or mean and the standard error (mean ± SE) of the serum sclerostin levels in any AS patients and HC. If there were duplicate publications, the one with the largest samples was selected and any meeting or conference abstracts were excluded. All analyses were based on previous published studies; thus no ethical approval and patient consent were required.

### 2.2. Data Extraction and Quality Assessment

Two researchers independently extracted data from all identified records according to the following criteria: first author's name, publication year, country of study, patient ethnicity, study type, number, age, mean ± SD, source of control, measurement, and *P* value of the estimated effects. When original important data were uncertain in identified articles, we mailed the corresponding author to obtain further details. Any discrepancy on data extraction was discussed by the two authors.

Methodological quality of each of the articles was also assessed and scored independently by the two researchers using the Newcastle-Ottawa quality assessment scale (NOS) for case-control study and Agency for Healthcare Research and Quality (AHRQ) for cross-control study. NOS is composed of eight questions with nine possible points: (1) participants selection, 0–4; (2) subjects comparability, 0–2; and (3) ascertainment for the exposure, 0–3. AHRQ consists of 11 items. An item would be scored “0” when it was answered with “NO” or “UNCLEAR”; if it was answered with “YES,” then the item would be scored “1”.

### 2.3. Statistical Analysis

To evaluate the overall serum sclerostin levels, we calculated the standardized difference (SMD) for every study with 95% confidence intervals (CIs) due to the fact that the units of concentration of serum sclerostin were different. The mean ± SD was extracted and calculated in any included publications. While the original data were mean ± SEM, we transformed them to mean ± SD. *I*^2^ statistic was calculated to assess heterogeneity for the outcomes. A value of 25%−50% indicates a low degree of heterogeneity, a value of 50%–75% indicates a moderate degree, and a value of >75% indicates a high degree. When *I*^2^ value was >50%, a random-effects model was used to pool the data; otherwise, a fixed-effects model was selected. The funnel plot was applied to estimate the publication bias. Sensitivity analysis was used to investigate the source of heterogeneity. *P* < 0.05 was considered statistically significant. All statistical analyses were carried out using RevMan 5.3 (the Cochrane Collaboration).

## 3. Results

### 3.1. Publication Search

**Figure 1:**
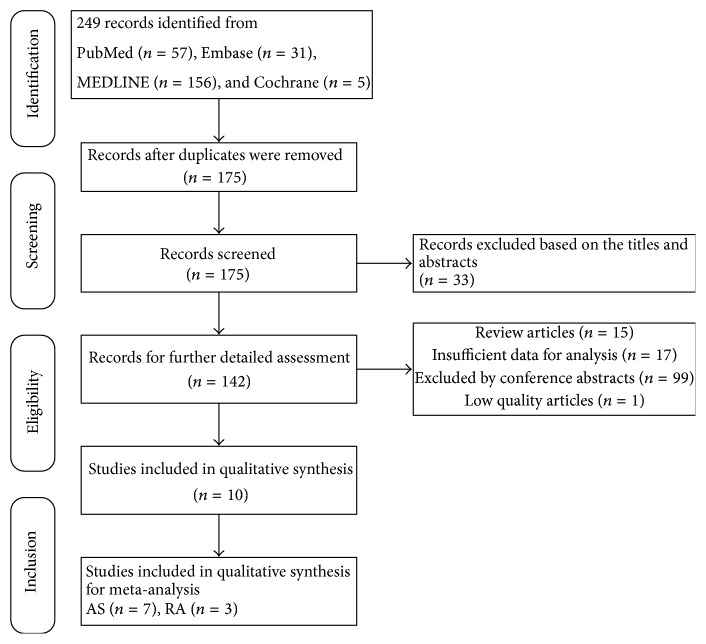
Study selection flow chart.

Initially altogether 249 articles were acquired. Among them, 57 articles were searched from PubMed, 156 from Embase, 31 from MEDLINE, and 5 from Cochrane Library. After reviewing the abstracts and full text, 239 articles were excluded due to their duplicate publication, unmatched purposes, review, conference abstracts, and low quality (Figure 1), and 10 articles which consisted of AS (*n* = 7) and RA (*n* = 3) were eventually included in this meta-analysis.

### 3.2. Characteristics of This Study

Finally seven studies [9, 12–17] including 420 AS patients and 317 HC and three studies [18–20] including 145 RA patients and 127 HC were in accordance with the inclusion criteria. The basic features of the included studies were shown in Table 1. The testing method of serum sclerostin levels in all the ten studies was enzyme-linked immunosorbent assay (ELISA). The results of methodological quality assessment using NOS for case-control studies and AHRQ for cross-sectional studies were shown in Table 1, in which the scores of the included articles were between 6 and 9.

### 3.3. Meta-Analysis in AS

**Figure 2:**
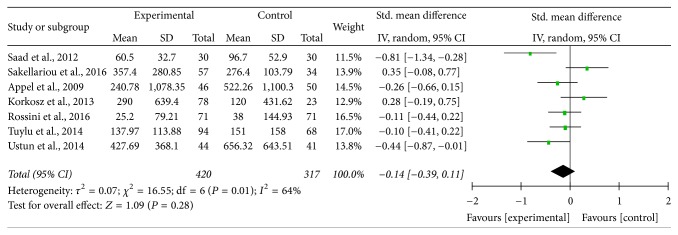
Forest plot of serum sclerostin levels for AS patients versus healthy controls.

**Figure 3:**
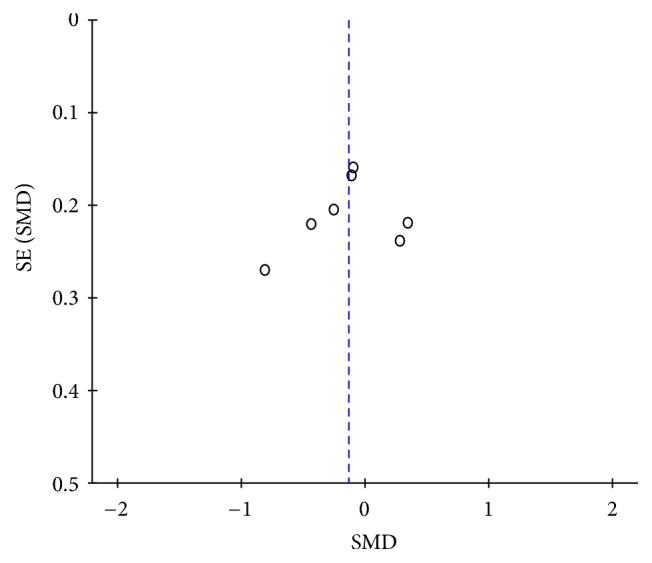
The shape of funnel plot.

**Figure 4:**
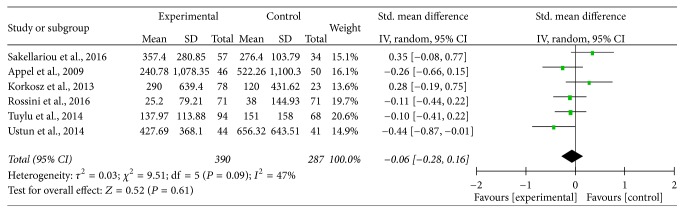
Forest plot of serum sclerostin levels for AS patients versus healthy controls, which excluded the study of Saad et al.

Among the seven studies, the heterogeneity was statistically significant (*P* = 0.01; *I*^2^ = 64%), and random-effects model was used, which showed that serum sclerostin levels in patients with AS were not statistically different compared with those in HC (SMD = −0.14; 95% CI = [−0.39,0.11]; *P* = 0.28) (Figure 2). The shape of the funnel plot, which was recommended for estimating the bias stated in Cochrane Handbook, looks to be symmetrical, indicating that potential publication bias might slightly affect the present meta-analysis (Figure 3). In order to investigate the source of heterogeneity, the sensitivity analysis was performed and it was found that the source of heterogeneity was mainly from the study of Carla GS Saad et al., which only recruited AS patients with a Bath AS Disease Activity Index (BASDAI) ≥ 4 and/or refractory high inflammatory parameters. This was a big difference from the other included studies. After excluding the data extracted from the study, the value of *I*^2^ was reduced to 47% (*P* = 0.09), which was considered to be acceptable, and the pooled SMD = −0.06; 95% CI = [−0.28,0.16]; *P* = 0.61 (Figure 4).

### 3.4. Meta-Analysis in RA

**Figure 5:**
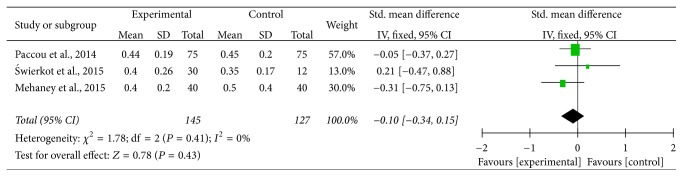
Forest plot of serum sclerostin levels for RA patients versus healthy controls.

Among the three studies of serum sclerostin in RA, the heterogeneity was not significant (*P* = 0.41; *I*^2^ = 0), and fixed-effects model was used, and it demonstrated that serum sclerostin levels between RA patients and HC were not significantly different (SMD = −0.10; 95% CI = [−0.34,0.15]; *P* = 0.43) (Figure 5). The shape of the funnel plot was not shown due to the fact that the sample is very small.

## 4. Discussion

Sclerostin had been considered to work as a suppressor during bone formation, which was backed up by the observation that the differentiation and proliferation of human and mouse osteoblastic cells were suppressed as exogenous sclerostin was added to the cultures [21–23]. Furthermore, the investigation of sclerostin knockout mice demonstrates striking increases in bone formation, bone mineral density, and bone strength [24]. The accumulating evidences showed an adverse impact of sclerostin during bone formation. The exact mechanism by which sclerostin affects the development of AS remains unclear. It is generally agreed that sclerostin inhibits the development of bone formation through the Wnt pathway [5, 25]. Sclerostin binds to LRP-5/LRP-6 and prevents Wnt proteins from reaching LRP-5/LRP-6 and gives rise to inhibiting the canonical Wnt signaling pathway [7, 8]. During the development, AS is characterized by excessive bone formation, like syndesmophytes and enthesiophytes [26]. Therefore, the declined sclerostin may contribute to the binding of Wnt proteins and LRP-5/LRP-6, promoting the Wnt signaling.

In this present study, we retrieved seven articles to estimate the serum sclerostin levels in AS patients by meta-analysis. These results demonstrated no difference in serum sclerostin levels between AS patients and HC, suggesting that serum sclerostin levels may be irrelevant to the pathogenesis of AS in patients. Dickkopf-1 (Dkk-1), another Wnt signal pathway antagonist, had been reported to increase in AS patients in a meta-analysis [27]. A recent study suggested that AS progress includes the cycles of bone resorption and formation [28]. Sclerostin and Dkk-1 may also serve as promoters in bone resorption. In addition to regulation of bone homeostasis, Wnt signaling pathway had been suggested to affect the T cell populations and behavior, which plays a center role in AS. Wnt signaling promotes differentiation of regulatory T cells with FOXP3 and inhibited differentiation of proinflammatory T cells such as T helper 1 (Th1) and Th17 cells [29, 30], indicating that sclerostin may emerge as a double-edged sword during bone formation.

Considering the suppression of sclerostin in bone formation, the role of sclerostin was also investigated in RA patients. Vis et al. had firstly shown that serum sclerostin level related to the disease activity and radiographic joint damage in RA patients; however, we only get the abstract of their study. Sclerostin inhibition is considered as a powerful tool to enhance bone repair in inflammatory arthritis in mice reported by Chen et al., indicating that sclerostin plays a pivotal role in the development of RA [31]. Recently, several studies [18–20] had demonstrated that serum sclerostin levels between RA patients and controls were not significantly different, and Mehaney et al. had further revealed that there was no significant correlation between serum sclerostin level and disease activity and bone mineral density. In our meta-analysis, we evaluated the association of serum sclerostin levels with RA, and there was no significant difference between RA patients and HC, indicating that sclerostin may not implicate the development of human RA.

As far as we know, this is the first comprehensive meta-analysis of serum sclerostin levels in AS and RA patients. However, several limitations should be considered in this study. First, there are very few studies after the first sorting, which account for just 4%, and the number of patients is relatively small; thus the limited size might affect the conclusion. Second, the articles, which only support median and range, were excluded. The method of transformation had been reported by Hozo et al. [32]; however, the result was not accurate as transformed and even presented the opposite results [33, 34]. Third, the information about factors that affected serum sclerostin was not given in the included studies, like age, sex, and ethnicity. Therefore, we will not succeed in further analysis between serum sclerostin and AS, which may influence the reliability of our study. Finally, the sclerostin enzyme-linked immunosorbent assay (ELISA) kit, which is different among the included papers, should be considered. In the group of included papers of AS, Saad et al., Rossini et al., and Tuylu et al. used Biomedica kit, Appel et al. and Korkosz et al. used R&D Systems kit, and Sakellariou et al. and Ustun et al. used BD Biosciences kit. In the group of included papers of RA, they all used TECOmedical kit. The serum sclerostin concentrations were higher when measured with the Biomedica kit as compared with TECOmedical kit and R&D Systems kit [35, 36]. This may be used to explain why the value of *I*^2^ was, respectively, high and low in the meta-analysis of serum sclerostin in AS patients and RA patients. To our knowledge, no data have been published comparing the BD Biosciences kit and R&D systems kit, Biomedica kit, or TECOmedical kit. The variability in values generated from these sclerostin ELISA kits raises questions regarding the accuracy and specificity of the assays.

In conclusion, our meta-analysis demonstrated that serum sclerostin levels in AS and RA patients were not significantly different from those in HC. These results suggest that sclerostin may not be associated with the development of AS and RA in patients. Nonetheless, determining the underlying mechanisms of sclerostin in AS and RA patients still awaits further analysis using larger samples.

## Figures and Tables

**Table 1:** Characteristics of included studies in the present meta-analysis.

				Case				Control								
References	Year	Region	Study type	*N*	Sex (M/F)	Mean (pg/ml)/(pmol/L)	SD (pg/ml)/(pmol/L)	*N*	Sex (M/F)	Mean (pg/ml)/(pmol/L)	SD (pg/ml)/(pmol/L)	*P*	Criteria for diseases	Source for control	Measurement	Quality score
Saad et al. [12]	2012	Brazil	Case-control	30	24/6	60.5	32.7	30	24/6	96.7	52.9	0.002	New York (1984)	Hospital	ELISA	6
Sakellariou et al. [13]	2016	Greece	Cross-sectional	57	53/4	357.4	280.85	34	32/2	276.4	103.79	0.611	New York (1984)	NA	ELISA	7
Appel et al. [14]	2009	German	Case-control	46	30/16	240.78	1078.35	50	33/17	522.26	1100.3	<0.01	NA	NA	ELISA	7
Korkosz et al. [9]	2013	Poland	Cross-sectional	78		290	639.4	23		120	431.62	0.09	New York (1984)	NA	ELISA	6
Rossini et al. [15]	2016	Italy	Case-control	71	59/12	25.2	79.21	71	59/12	38	144.93	<0.01	New York (1984)	Hospital	ELISA	8
Tuylu et al. [16]	2014	Turkey	Case-control	94	65/29	137.97	113.88	68	48/20	151	158	0.6	New York (1984)	NA	ELISA	6
Ustun et al. [17]	2014	Turkey	Cross-sectional	44	34/10	427.69	368.1	41	31/10	656.32	643.51	0.037	New York (1984)	NA	ELISA	7
Paccou et al. [18]	2014	France	Cross-sectional	75	19/56	440	190	75	19/56	450	200	0.96	ACR (2010)	Hospital	ELISA	8
Mehaney et al. [19]	2015	Egypt	Cross-sectional	40	28/12	400	200	40	28/12	500	200	0.14	ACR (2010)	Hospital	ELISA	8
Świerkot et al. [20]	2015	Poland	Clinical study	30	30/0	400	260	12	12/0	350	170	>0.05	ACR (1987)	NA	ELISA	—

NA: not available; SD: standard deviation.
