# Corrigendum to “Serum Sclerostin Levels in Patients with Ankylosing Spondylitis and Rheumatoid Arthritis: A Systematic Review and Meta-Analysis”

**DOI:** 10.1155/2017/3953474

**Published:** 2017-10-17

**Authors:** Jianfeng Shi, Haijian Ying, Juping Du, Bo Shen

**Affiliations:** ^1^Department of Clinical Laboratory, Taizhou Hospital of Zhejiang Province, Affiliated Hospital of Wenzhou Medical University, Taizhou, Zhejiang Province, China; ^2^Wenzhou Medical University, Wenzhou, Zhejiang Province, China

In the article titled “Serum Sclerostin Levels in Patients with Ankylosing Spondylitis and Rheumatoid Arthritis: A Systematic Review and Meta-Analysis” [1], the affiliation listed for the first author was incorrect. The corrected authors' list and affiliations are shown above.
